# The Treatment of Diphtheria with Antitoxin

**Published:** 1895-02

**Authors:** A. Smirnow, F. C. Moehlau

**Affiliations:** 1343 Jefferson Street; Buffalo, N. Y.


### THE TREATMENT OF DIPHTHERIA WITH ANTITOXIN.

### TO BE PREPARED WITHOUT THE MEDIATION OF THE ANIMAL ORGANISM.

By. DR. A. SMIRNOW.

Translated from *Berl. Klin. Wochenschr.*, xxxi., 30, 1894.

By F. G. MOEHLAU, M. D., of Buffalo, N. Y.

SMIRNOW expresses himself as much reserved as to the practical value of the serum therapy. He discusses the question, whether the processes of antitoxin formation are purely vitalistic or whether they occur according to known chemical laws!

Dr. Smirnow, induced by Professor Nenki, made experiments to investigate whether it is not possible to produce certain properties in serum of normal or diseased animals by oxidation, or reducing reaction such as are peculiar to animals made artificially immune by antitoxin. After several unsatisfactory results, Smirnow began to subject the serum of dogs to an electrolysis. He observed, at first, cloudiness on the negative pole and coagulation which redissolved again, and that on the positive pole the fluid remained clear. If the acid liquid derived from the negative pole, or the alkaline liquid from the positive pole, were injected below the skin of a rabbit, no noticeable change occurred, whereas, after the injection of 1 c. cm. of the decomposed and neutralized serum, (no matter whether it be derived from the negative or positive pole,) a decided rise in temperature was noticed, slowly decreasing. Further investigations showed that this action is dependent upon changes in the serum albumin, not the globulin. Animals which had been infected with glanders, diphtheria, lyssa, etc., and to all of which said serum had been injected, showed the rise in temperature but died of the typical disease.

Smirnow prepared new diphtheria cultures in serum, solution of albumin and globulin, and he was able to obtain diphtheriatoxin of the first two species of cultures, which he subjected to electrolysis and found that by this method the bacilli are not destroyed. With the first experiment it became evident that it was possible for the toxin to be changed into antitoxin by means of electrolysis. Smirnow was hereby enabled to cure diphtheria in rabbits by injecting electrolitic serum and albumin. Since then, Smirnow has succeeded in preparing antitoxin by electrolyzing cultures in bouillon *with positive success.* The activity and strength of the antitoxin is relative in proportion to the virulence of the toxin subjected to electrolysis. Smirnow is of the opinion that by his method a more active immunizing agent may be prepared than by the inoculation method. He promises further investigations in this line, especially also in tuberculosis.

1343 JEFFERSON STREET.

---

DOCTORS should be careful about buying preparations labelled "antitoxin." The temptation to manufacturing chemists is, of course, great, to offer this promising remedy for sale, but inasmuch as the process of manufacture requires several months, there is as yet no reliable antitoxin of American make. Thus far only the German or French preparation can be considered genuine or effective.—*Atlanta Medical and Surgical Journal.*

